# LINC00680 enhances hepatocellular carcinoma stemness behavior and chemoresistance by sponging miR-568 to upregulate AKT3

**DOI:** 10.1186/s13046-021-01854-5

**Published:** 2021-01-26

**Authors:** Gege Shu, Huizhao Su, Zhiqian Wang, Shihui Lai, Yan Wang, Xiaomeng Liu, Luo Dai, Yin Bi, Wei Chen, Weiyu Huang, Ziyan Zhou, Songqing He, Hongliang Dai, Bo Tang

**Affiliations:** Department of Hepatobiliary Surgery, Key Laboratory of Basic and Clinical Application Research for Hepatobiliary Diseases, The First Affiliated Hospital of Guangxi Medical University, No. 6 Shuangyong Road, Nanning, 530021 Guangxi People’s Republic of China

**Keywords:** Hepatocellular carcinoma, LINC00680, miR-568, AKT3, Stemness, Chemosensitivity

## Abstract

**Background:**

Hepatocellular carcinoma (HCC) has an extremely poor prognosis due to the development of chemoresistance, coupled with inherently increased stemness properties. Long non-coding RNAs (LncRNAs) are key regulators for tumor cell stemness and chemosensitivity. Currently the relevance between LINC00680 and tumor progression was still largely unknown, with only one study showing its significance in glioblastoma. The study herein was aimed at identifying the role of LINC00680 in the regulation HCC stemness and chemosensitivity.

**Methods:**

QRT-PCR was used to detect the expression of LINC00680, miR-568 and AKT3 in tissue specimen and cell lines. Gain- or loss-of function assays were applied to access the function of LINC00680 in HCC cells, including cell proliferation and stemness properties. HCC stemness and chemosensitivity were determined by sphere formation, cell viability and colony formation. Luciferase reporter, RNA immunoprecipitation (RIP), and RNA pull-down assays were performed to examine the interaction between LINC00680 and miR-568 as well as that between miR-568 and AKT3. A nude mouse xenograft model was established for the in vivo study.

**Results:**

We found that LINC00680 was remarkably upregulated in HCC tissues. Patients with high level of LINC00680 had poorer prognosis. LINC00680 overexpression significantly enhanced HCC cell stemness and decreased in vitro and in vivo chemosensitivity to 5-fluorouracil (5-Fu), whereas LINC00680 knockdown led to opposite results. Mechanism study revealed that LINC00680 regulated HCC stemness and chemosensitivity through sponging miR-568, thereby expediting the expression of AKT3, which further activated its downstream signaling molecules, including mTOR, elF4EBP1, and p70S6K.

**Conclusion:**

LINC00680 promotes HCC stemness properties and decreases chemosensitivity through sponging miR-568 to activate AKT3, suggesting that LINC00680 might be a potentially important HCC diagnosis marker and therapeutic target.

**Supplementary Information:**

The online version contains supplementary material available at 10.1186/s13046-021-01854-5.

## Background

Hepatocellular carcinoma (HCC) is one of the major cancers, ranking the third most frequent cause of cancer mortality worldwide [1, 2]. Despite significant improvement in surgical procedure and chemical therapy, the prognosis of HCC is still unsatisfactory. Population-based researches pointed out that the death rate of HCC continued to approach the incidence rate level, with a dismal 5-year survival rate of 14–18% [3]. Accumulative studies suggest that HCC exhibits high recurrence rate and chemotherapeutic resistance, leading to the high death rate of this malignancy [4, 5]. Therefore, it is urgent to uncover the molecular mechanism underlying HCC relapse and chemo-resistance, so as to find more effective therapeutic target and thereby further improve HCC prognosis.

Long non-coding RNAs (lncRNAs) are a type of RNA molecules with length > 200 nucleotides with no or feeble protein-coding functions [6]. Studies have shown that lncRNAs function as critical tumor regulators in a variety of cancers, implicated in multiple biological processes, such as cell growth, differentiation, migration, and cancer stemness, etc. [7–9]. Mechanically, it has been revealed that lncRNAs either act as molecular sponges for microRNAs (miRNAs), thereby preventing miRNA-mediated suppression of downstream target gene expression (cytosolic lncRNAs), or serving as functional scaffolds recruiting regulatory proteins to their target chromosomal regions (nuclear lncRNAs) [4, 10, 11]. LINC00680 is a newly identified lncRNA molecule and its role in cancer progression is largely unknown, with only one existing report suggesting LINC00680 as a tumor promoter in glioblastoma [12].

In the current study, we systematically examined the expression change of LINC00680 in HCC tissues as contrast to corresponding adjacent non-tumor tissues and determined its correlation with clinical pathological parameters and patient prognosis. We then explored the functional roles of LINC00680 in HCC cell stemness and chemo-resistance in vitro and in vivo. Our results highlight the novel role of LINC00680 as a metastasis-promoting molecule in HCC by sponging miR-568 and upregulating AKT3, implying a novel potential diagnostic and therapeutic target for HCC.

## Methods

### Patient samples

A total of 74 pairs of HCC cancerous and corresponding non-cancerous tissues were gathered from HCC patients who underwent surgery from 2017 to 2020 at The First Affiliated Hospital of Guangxi Medical University. These tissue samples were immediately transferred into liquid nitrogen right after the surgery and then stored at − 80 °C for future use. This study was reviewed and approved by the ethics committee of The First Affiliated Hospital of Guangxi Medical University, and written informed consent in accordance with the Declaration of Helsinki and its later revision was provided by all the patients.

### Cell lines and cell culture

HCC cell lines SNU-449, SNU-182, Huh7, LM3, Bel-7405, SK-hep1, Hep3B and normal human liver cell line L02 were purchased from the American Type Culture Collection (Manassas, VA, USA) or Institute of Biochemistry and Cell Biology (Chinese Academy of Sciences, Shanghai, China). SNU-449, SK-hep1 and SNU-182 were cultured in RPMI-1640 medium (Gibco, USA) containing 10% fetal bovine serum (FBS, Gibco). The other cells were cultured in high-glucose Dulbecco’s modified Eagle medium (DMEM) (Gibco) containing 10% FBS. Penicillin (100 U/ml) and streptomycin (100 μg/ml) were supplemented to the medium to reduce the chance of microbial contamination. All the cells were maintained in a humidified atmosphere at 37 °C with 5% CO_2_/95% air.

### Cell transfection

Short hairpin RNA (shRNA) for LINC00680 knockdown and its negative control were obtained from GeneChem (Shanghai, China). pcDNA3.1 (+) vector for LINC00680 overexpression and its corresponding control, miR-568 mimics, miR-568 inhibitor and their negative controls, AKT3-targeted siRNA, pcDNA3.1 (+) vector for AKT3 overexpression, and their corresponding negative controls were all synthesized by GenePharma (Shanghai, China). Cells were seeded on a six-well plate at a density of 5 × 10^5^ cells /well. Transfection operation began after 24 h incubation at 37 °C in a humidified incubator. All the in vitro transfections were performed using Lipofectamine TM 3000 Reagent (Thermo Fisher Scientific) according to the manufacturer’s instructions. Lentiviral infection method was used to deliver sh-LINC00680 into HCC cell lines used for in vivo mouse xenograft tumor model.

### Quantitative real time polymerase chain reaction (qRT-PCR)

Total RNA were extracted from cells and tissues by RNA simple Total RNA Kit (TIANGEN, DP419). The reverse transcription of cDNA was carried out by Revert Aid First Strand cDNA Synthesis Kit (Thermo Scientific, #K1622) and poly (A) polymerase Reaction Buffer (NEB, M0276s) according to the manufacturer’s instructions. QRT-PCR was performed by iTaqTM Universal SYBR® Green Supermix. The fold change of RNA expression was quantified using the 2^-ΔΔCt^ method after normalizing with GAPDH or U6 expression. The primers used to detect miR-568 were designed by GeneCopoeia with their sequences protected by a patent (http://www.igenebio.com/tech/datasheet/index.php). The mRNA Primers used are listed in Table S1.

### Western blotting

Protein samples were extracted using cold RIPA lysis buffer containing protease inhibitor cocktail (Roche, Mannheim, Germany), with protein concentration determined by the BCA method. Equal amounts of protein lysates were run on a sodium dodecyl sulfate polyacrylamide gel electrophoresis (SDS-PAGE) Bio-Rad system. Separated proteins were detected with the corresponding antibodies and visualized using enhanced chemiluminescence (ECL). The primary antibodies included those against AKT3, p-AKT3, mTOR, p-mTOR, elF4EBP1, p-elF4EBP1, p70S6K, p-p70S6K, CD133, OCT4, NANOG, SOX-2 and β-actin. All these antibodies were purchased from Abcam Corporation (Cambridge, MA, USA).

### Sphere formation assay

The Cells in exponential growth phase were seeded in ultralow attachment six well plates (Corning) at a density of 1000 cells/well and cultured for 1 week (Primary spheres) in DMEM/F12 medium (Invitrogen, Shanghai, China) supplemented with B27 (1:50, GIBCO, Shanghai, China), 20 ng/ml EGF (Sigma, Shanghai, China), and 20 ng/ml bFGF (Sigma, Shanghai, China). Subsequently, the primary spheres were collected, dissociated with trypsin and plated again to generate secondary spheres through the same process. Finally, the number of the spheres was counted a microscope.

### Cell proliferation assay

Cell count kit-8 (CCK8, Dojindo, Japan) and colony formation assay were used to determine cell proliferation. For CCK8 assay, cells were seeded in 96-well plates at a density of 1 × 10^3^ cells/well, grew overnight, and the treated with different concentration of 5-Fu for 48 h. Subsequently, 10 μl CCK-8 solution was added to each well and incubated for another 2 h at 37 °C under a humidified 5% CO_2_/95% air atmosphere. Absorbance values were measured on a microplate reader (spectramax plus384, Molecular Devices, USA) at 450 nm. For the colony-formation assay, cells were seeded in 6-well plates at a density of 1000 cells/well, and grew for 7 days with or without 5-Fu. After rinsed twice by PBS, formed cell colonies were fixed in 70% methanol for 30 min and stained with 0.5% crystal violet for 30 min. The cell colonies larger than 50 cells were counted for comparison between groups.

### Analysis of the tumor-forming potential in vivo

6-week-old male BALB/c nude mice (Shanghai Slac Laboratory Animal Co. Ltd., China) were obtained and bred under a SPF (specific pathogen-free) condition. LINC00680 stably downregulated SNU-449 and SK-hep1 cells and their negative controls were subcutaneously injected into the mice (5 mice/group). The volume of tumors was continuously measured using a Vernier caliper. Five weeks later, the mice were euthanatized to measure the tumor weight. The animal experimental protocols were in accordance with institutional guidelines approved by the Animal Care and Use Committee.

### Dual luciferase reporter assay

The sequences of wild- or mutant-type AKT3 3′-UTR were inserted into PmiRGLO dural-luciferase reporters. Thereafter, the recombinant plasmids were co-transfected with miR-568 mimics or miRNA mimics negative control (NC) into 293 T cells by lipofectamine TM 3000 (Thermo Fisher). After 36 h transfection, Luciferase assay system (Promega, Madison, USA) was used to determine the relative luciferase activity normalizing to renilla luciferase activity. The binding between LINC00680 and miR-568 was verified using a similar method.

### RNA immunoprecipitation (RIP) assay

We used Magna RIP™ RNA-Binding Protein Immunoprecipitation Kit (Millipore, USA) to perform RIP experiments according to the manufacturer’s instructions. Beads were incubated with AGO2 antibodies and washed with wash buffer, the complexes were then incubated with 0.1% SDS/Proteinase K (0.5 mg/ml, 30 min at 55 °C) to remove proteins, LINC00680 and miR-568 were detected by qRT-PCR. The supernatant of RIP lysate was used to test the expression of AGO2 by western blot.

### RNA pull-down assay

Pierce™ RNA 3′ End Desthiobiotinylation Kit (Thermo, 20,163) was used to label LINC00680 for attachment to streptavidin magnetic beads which can capture protein complex combined with labeled LINC00680. According to the manufacturer’s instructions, Thermo Scientific Pierce Magnetic RNA-Protein Pull-Down Kit (Thermo, 20,164) was used to perform RNA pull-down assay. AGO2 was assessed by western blot, and LINC00680 and miR-568 were detected by qRT-PCR.

### Microarray analysis

We performed a microRNA microarray analysis using SNU-449 cells transfected with miR-568 mimics or its negative control. Total RNA were extracted by RNA simple Total RNA Kit (TIANGEN, DP419) according to the manufacturer’s instructions. NanoDrop one (Thermo Fisher Scientific) was used for measurement of RNA quantity. Microarray analysis were performed by Huayin Health Medical Group Co, Ltd. (Guangzhou, China). These obtained data were then subjected to KEGG and GO analysis. Cluster 3.0 and Java Tree View software were used to visualize heat maps.

### Statistical analysis

Data were presented as the mean ± standard deviation (SD) from at least three independent experiments and analyzed using SPSS 24.0 for Windows (SPSS, Chicago, IL, USA). Student’s t test or one-way analysis of variance (ANOVA) plus Tukey’s post-hoc test was used for comparisons between groups. Kaplan-Meier method was used for analysis of overall survival (OS) and disease-free survival (DFS). *P*-values < 0.01 were considered statistically significant.

## Results

### LINC00680 is upregulated in HCC, and predicts poor prognosis of HCC patients

**Fig. 1:**
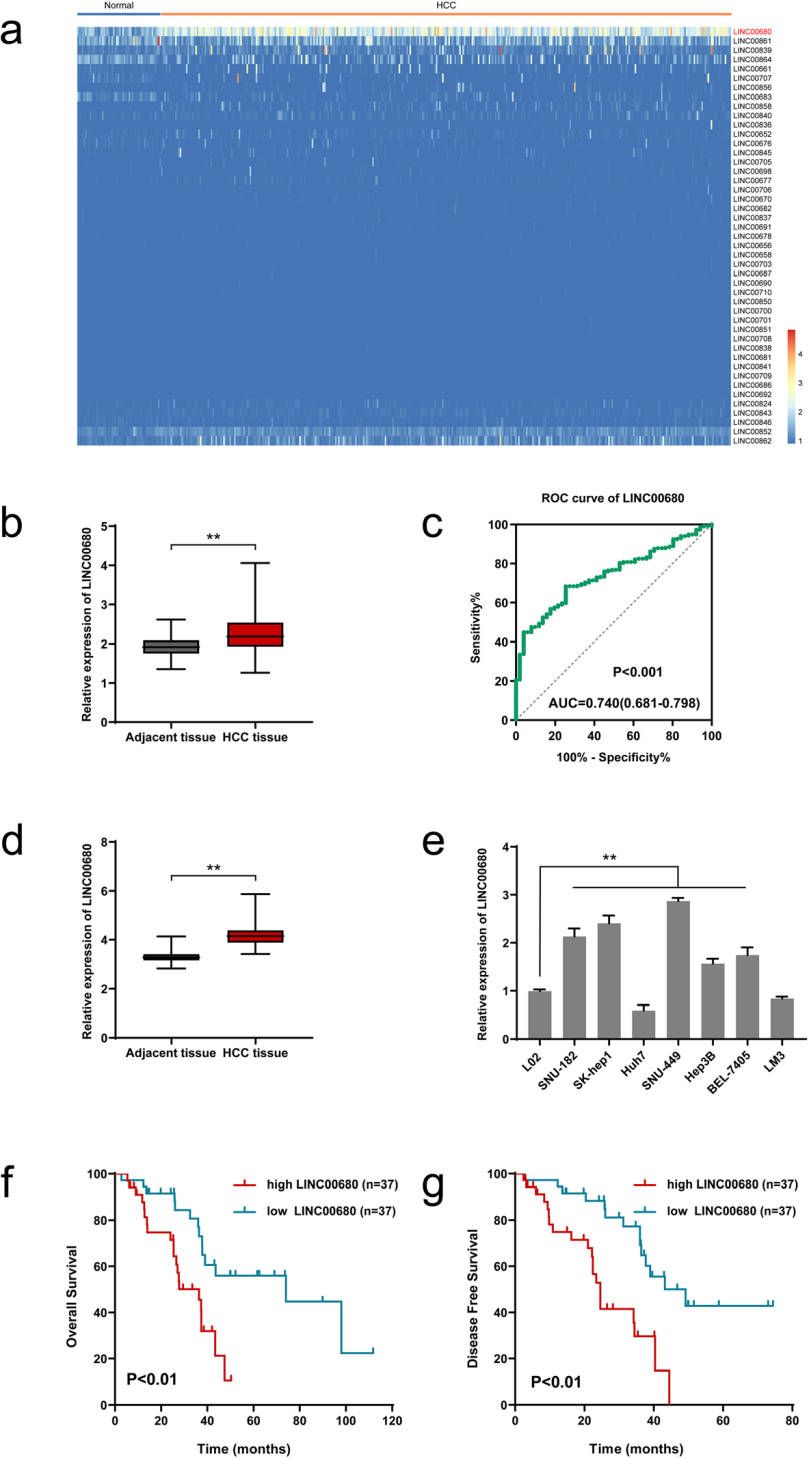
LINC00680 is upregulated in HCC, predicting poor prognosis of HCC patients. **a**. The heatmap of the differentially expressed LncRNAs between adjacent normal liver tissues (51 cases) and HCC tissues (370 cases) across TCGA database. **b**. Comparison of LINC00680 expression between 370 HCC tissues and 51 adjacent normal liver tissues based on TCGA dataset. **c**. The receiver operator characteristic (ROC) analysis determining the diagnostic value of LINC00680 expression level in differentiating between normal and malignant hepatic tissues using data from TCGA LIHC dataset. **d**. Expression of LINC00680 between 74 pairs of HCC tumor tissues and adjacent normal liver tissues. **e**. Relative expression of LINC00680 in HCC cell lines and L02 cells. **f**, **g**. Kaplan-Meier analyses for the correlation between the LINC00680 level and HCC prognoses in 74 patients (f. Overall survival; **g**. Disease free survival). ^**^P < 0.01

We first compared the expression levels of LncRNAs between normal liver tissues (51 cases) and HCC tissues (370 cases) across TCGA database. The results showed that LINC00680 was on the top list among the differentially expressed lncRNAs, namely LINC00680 was significantly upregulated in HCC tissues (Fig. 1a-b). Furthermore, the receiver operator characteristic (ROC) analysis showed that LINC00680 expression level had significant diagnostic value in discriminating between HCC and adjacent healthy tissues (Fig. 1c). To confirm these bioinformatic data, we further compared the expression levels of LINC00680 in 74 paired HCC tissues and corresponding adjacent non-tumor tissue by qRT-PCR. As is shown in Fig. 1d, we found that LINC00680 was significantly upregulated in the HCC specimens. Additionally, LINC00680 expression was significantly correlated with tumor stage, tumor size, cirrhosis, and microvascular invasion (Table 1). We also examined the expression levels of LINC00680 in L02, SNU-182, SK-hep1, Huh7, SNU-449, Hep3B, BEL-7405, and LM3 cell lines, and found a remarkably higher expression of LINC00680 in five out of all the seven HCC lines (SNU-182, SK-hep1, SNU-449, Hep3B, and BEL-7405) than that in the normal liver cell line (L02) (Fig. 1e). Kaplan-Meier survival analysis confirmed that patients with high level of LINC00680 exhibited poorer prognosis both in overall survival (Fig. 1f) and disease free survival (Fig. 1g). These data indicate that LINC00680 may have an oncogenic role in HCC.

**Table 1:** Relationship between LINC00680 and clinicopathological parameters of 74 HCC patients

Variables	Number of cases	LINC00680 expression		*P* value
		low	high	
Ages				
≥ 50	40	21	19	
< 50	34	16	18	0.641
Gender				
Male	61	31	30	
Female	13	6	7	0.588
Etiology				
No	33	16	17	
Yes	41	21	20	0.815
Serum AFP				
≤ 200	38	18	20	
> 200	36	19	17	0.642
Tumor stage				
I/II	52	30	22	
III/IV	22	7	15	**0.042**
Tumor size				
≤ 5 cm	41	29	12	
> 5 cm	33	8	25	**< 0.001**
Alcohol history				
No	37	18	19	
Yes	37	19	18	0.816
Cirrhosis				
No	34	26	8	
Yes	40	11	29	**< 0.001**
Microvascular invasion				
No	41	30	11	
Yes	33	7	26	**< 0.001**
Smoking				
No	42	22	20	
Yes	32	15	17	0.639

### LINC00680 promotes HCC cell stemness and increase HCC chemoresistance both in vitro and in vivo

**Fig. 2:**
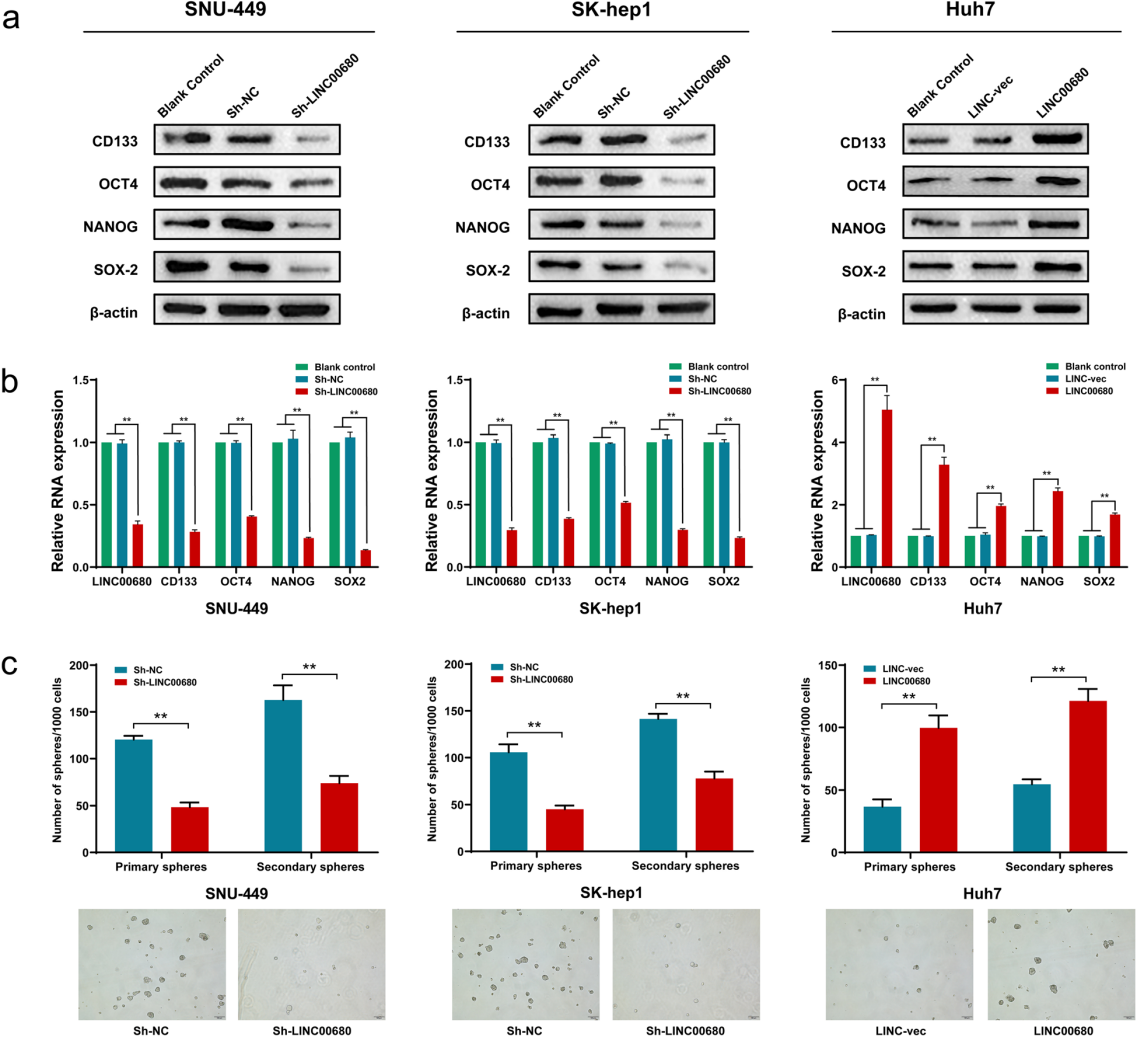
The LINC00680 regulates stemness properties in HCC cells. **a**, **b**. Analysis of the level of stemness-associated genes, including CD133, OCT4, NANOG and SOX-2 in LINC00680 knockdown SNU-449 and SK-hep1 cells, and LINC00680 overexpressed Huh7 cells by Western blot (**a**) and qRT-PCR (**b**), respectively. **c.** Statistical analysis of the primary and secondary sphere formation capacities in LINC00680 knockdown SNU-449 and SK-hep1 cells, and LINC00680 overexpressed Huh7 cells (upper panel) with the representative images showing secondary sphere formation in these cells (lower panel). ^**^P < 0.01

To explore the potential relevance of LINC00680 in the tumorigenesis of HCC, loss-of-function assays were performed in SNU-449 and SK-hep1 cell lines, and gain-of-function assays in Huh7 cell line. As seen in Fig. 2a-b, the stemness-associated markers, including CD133, OCT4, NANOG, SOX-2 were all significantly downregulated upon LINC00680 silencing. In contrast, LINC00680 overexpression triggered significant upregulation of stemness markers. Suppression of LINC00680 significantly inhibited primary and secondary sphere formation capability of SNU-449 and SK-hep1 cell, whereas LINC00680 overexpression in Huh7 cells produced an opposite result (Fig. 2c).

**Fig. 3:**
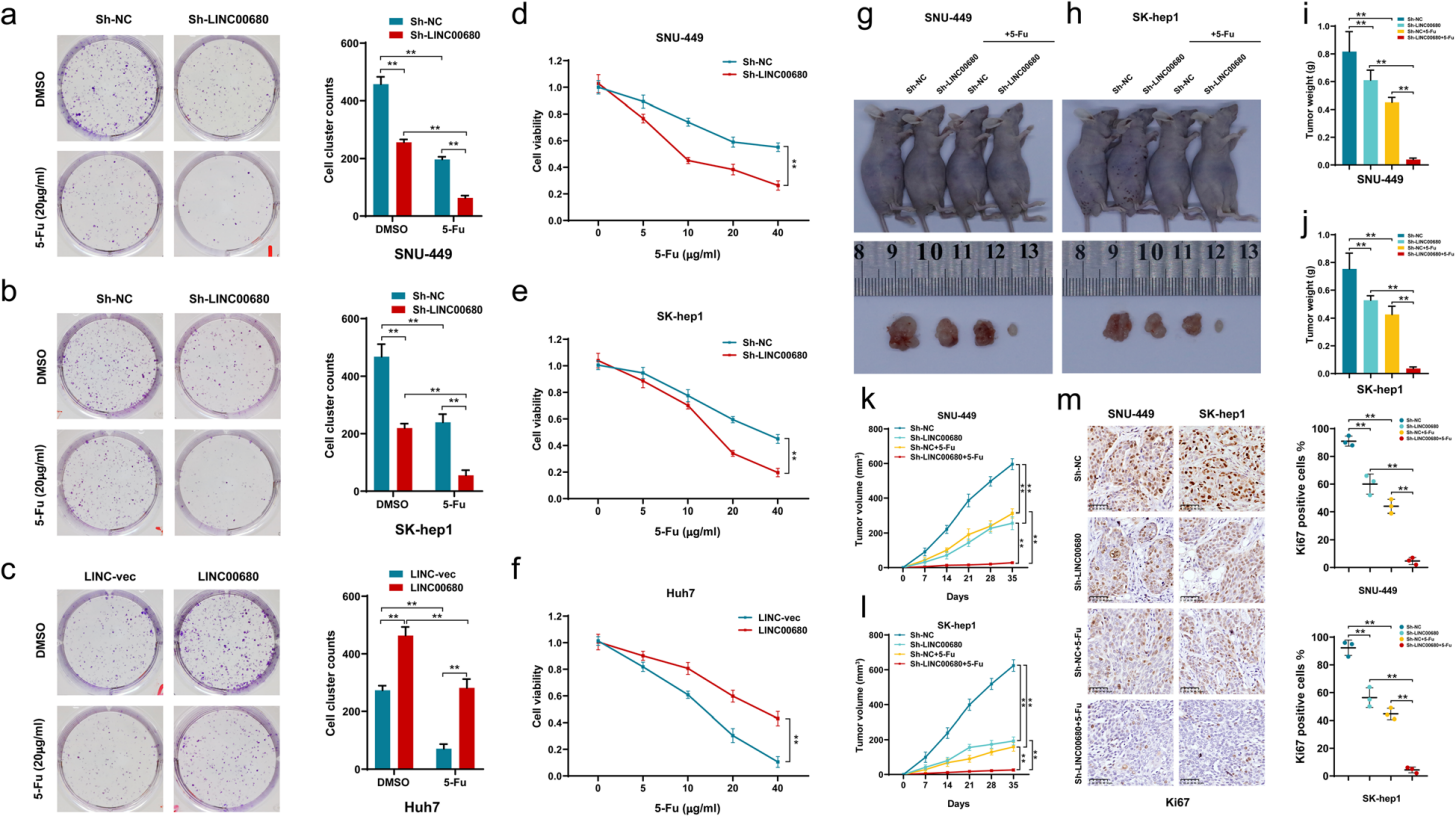
LINC00680 promotes HCC chemoresistance to 5-Fu in vitro and in vivo. **a**, **b**. Colony formation of7 sh-LINC00680-transfected cells after treatment with 5-Fu. **c.** Colony formation of LINC00680 overexpression plasmid-transfected cells after treatment with 5-Fu. **d**, **e**. Cell viability analysis using CCK8 in sh-LINC00680-transfected SNU-449 and SK-hep1 cells under different concentrations of 5-Fu. **f**. Cell viability analysis using CCK8 in LINC00680 overexpression plasmid-transfected Huh-7 cells under different concentrations of 5-Fu. **g**, **h**. Representative images of xenograft tumors of shLINC0068-transfected HCC cells in nude mice following intraperitoneal injection of 5-Fu. **i**, **j**. Tumor weight of sh-LINC00680- or sh-NC-transfected cells in nude mice after treatment with 5-Fu. **k**, **l**. Tumor growth curves of sh-LINC00680- or sh-NC-transfected cells in nude mice after treatment with 5-Fu during the indicated days. **m**. Ki67 expression in sh-LINC00680- or sh-NC-transfected cells in nude mice after treatment with 5-Fu. ^**^P < 0.01

Accumulating evidences suggest that chemosensitivity of tumor cells is closely related to their stemness characteristics [4, 13, 14]. 5-Fluorouracil (5-Fu) has long been used as a classic and highly effective chemotherapeutic agent in the treatment of multiple cancers, including HCC. However, clinically the majority of HCC patient exhibit primary and/or acquired chemoresistance to 5-Fu, greatly hindering its clinical applications in HCC [15]. For this, we next examined the potential involvement of LINC00680 in HCC sensitivity to 5-Fu, in vitro and in vivo. In the in vitro colony formation and CCK-8 assays, it was found that knockdown of LINC00680 significantly sensitized HCC cells to 5-Fu. However, LINC00680 ectopic overexpression produced a noticeable HCC chemoresistance against 5-Fu (Fig. 3a-f). In the in vivo experiments using the nude mouse xenograft model, we found that silencing LINC00680 remarkably enhanced the anti-tumor potential of 5-Fu (Fig. 3g-h). Our data showed that the inhibitory efficiency of 5-Fu on the xenograft weight (Fig. 3i-j) and volume (Fig. 3k-l) were both strengthened by LINC00680 knockdown. Consistently, the combination of Sh-LINC00680 with 5-Fu produced a stronger decrease of the expression of proliferation marker ki67, when compared with any single intervention (Fig. 3m). As expected, the stemness-related markers were modified following LINC00680 knockdown but not further affected by 5-Fu (Supplementary Figure S1a, b).

### LINC00680 sponges and down-regulates miR-568 level in HCC via the ceRNA mechanism

**Fig. 4:**
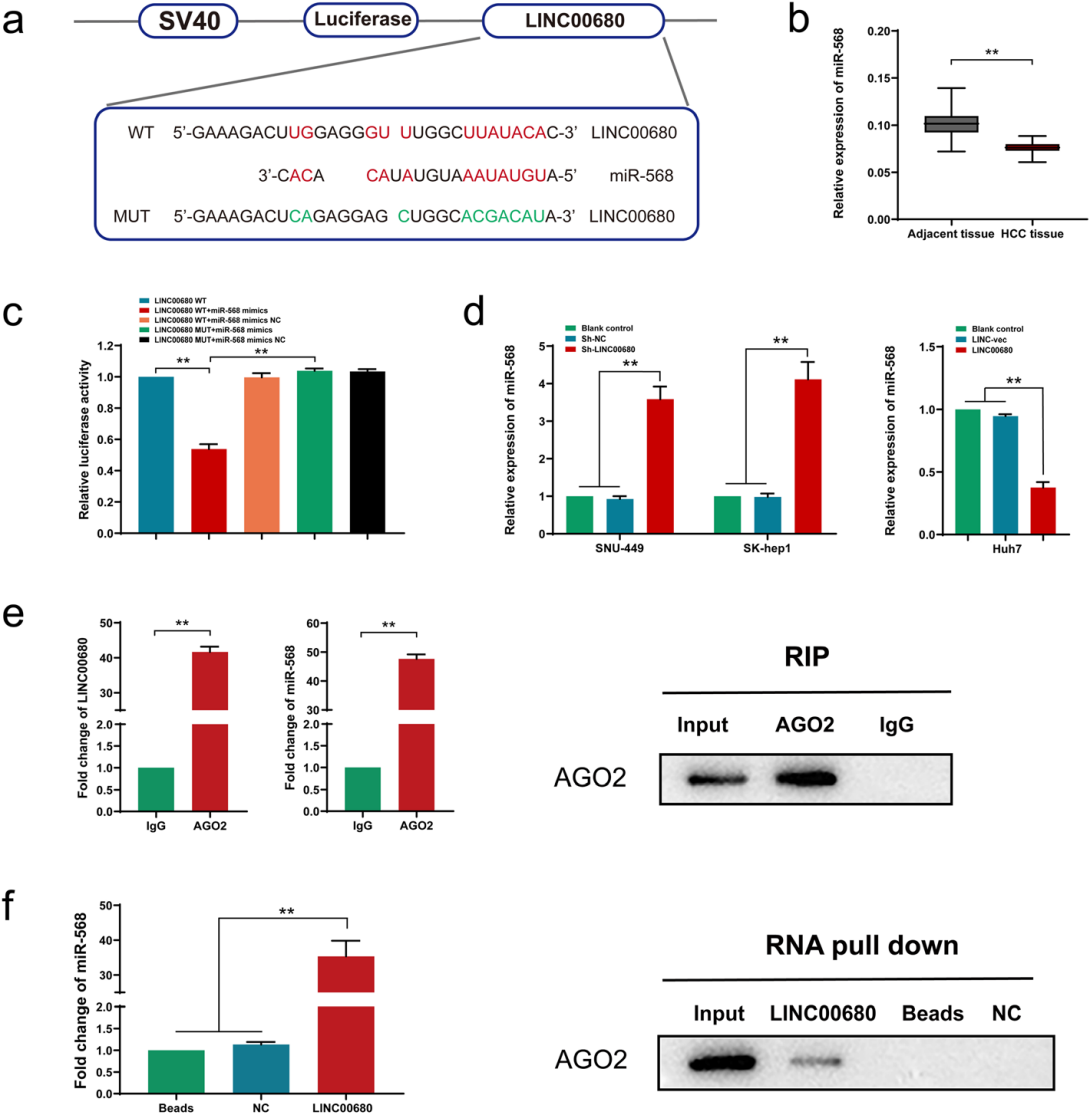
LINC00680 sponges and downregulates miR-568 in HCC cells. **a**. Conjectured binding sites between LINC00680 and miR-568 by LncBase. **b**. Comparison of miR-568 level between 74 pairs of HCC tissues and adjacent normal liver tissues. **c**. The relative luciferase activities in 293 T cells following the indicated transfection. **d**. Expression of miR-568 after LINC00680 manipulation. **e.** RIP assay for the relative enrichment of LINC00680 and miR-568 in anti-IgG- or anti-AGO2 specific immunoprecipitates. **f**. RNA pull-down assay was used to detect the interaction between LINC00680, miR-568 and AGO2. ^**^P < 0.01

LncRNA can function as a miRNA sponge and upregulate target gene expression via the competitive endogenous RNA (ceRNA) mechanism. Using bioinformatics database LncBase Predicted v.2, we identified a potential binding between LINC00680 and miR-568 sequences (Fig. 4a). Notably, a significant lower expression of miR-568 was also seen in HCC tissues than that in adjacent non-tumor tissues (Fig. 4b). Dual luciferase reporter gene assay showed a decreased firefly luciferase activity after co-transfection with wild-type (WT) LINC00680 vector and miR-568 mimics, which was not seen in cells co-transfected with mutant-type (MT) LINC00680 and miR-568 mimics (Fig. 4c). Additionally, miR-568 expression was significantly increased in LINC00680 knockdown SNU449 and SK-hep1 cell lines, whereas miR-568 level was remarkably reduced in LINC00680 overexpressed Huh7 cells (Fig. 4d). Therefore, we reckoned that miR-568 might be sequestered and negatively regulated by LINC00680, which would hinder RISC-mediated downstream mRNA silencing. To address this possibility, we further performed RIP and RNA pull-down assays. As shown in RIP assay, compared with IgG control, a much more enrichment of LINC00680 and miR-568 in Ago2 precipitated pellet (Fig. 4e). This result was further confirmed by the subsequent RNA pull-down assay (Fig. 4f). Collectively, these data suggested a molecular sponge role of LINC00680 for miR-568 via the ceRNA mechanism.

### MiR-568 targets to AKT3 in HCC

**Fig. 5:**
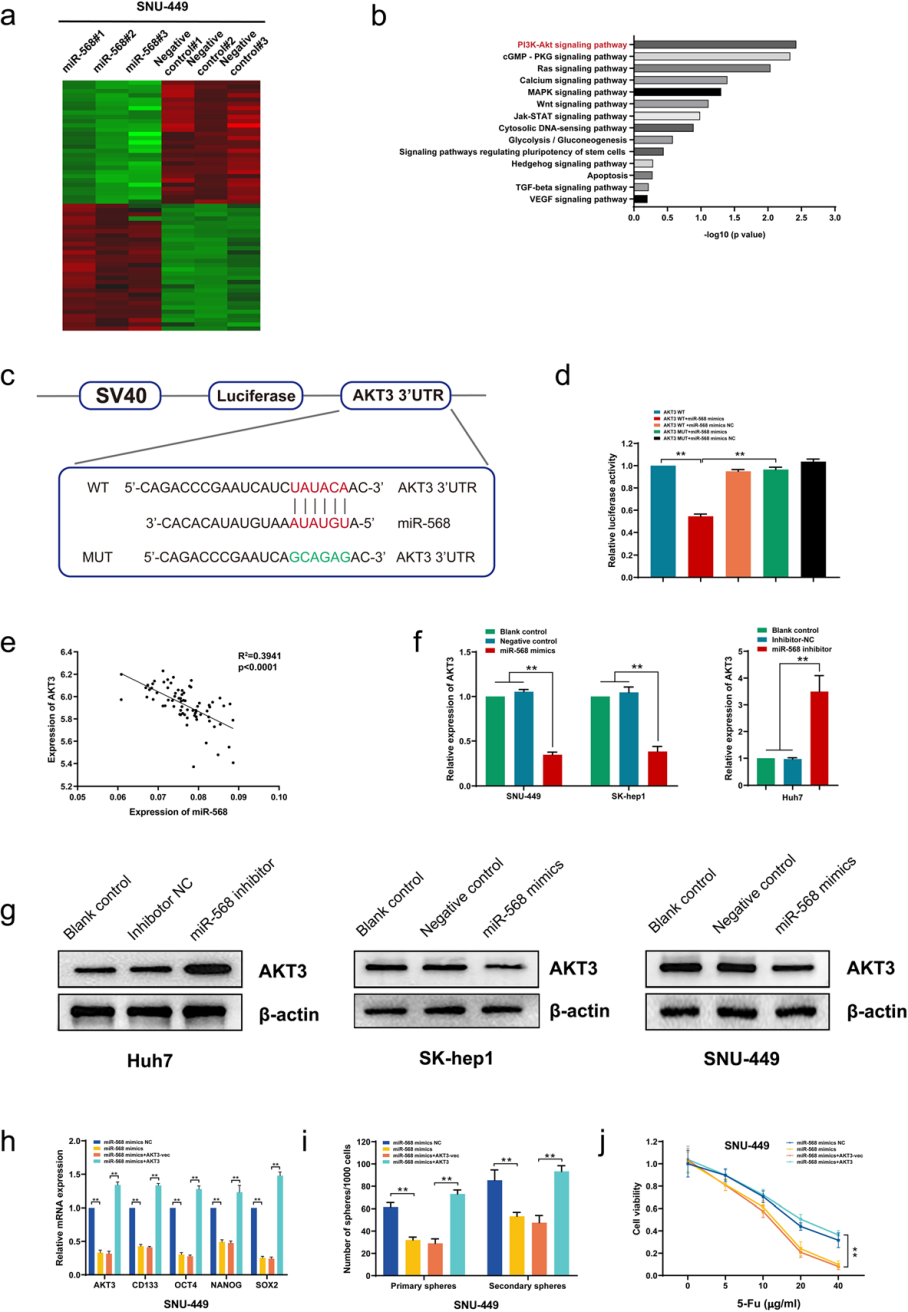
MiR-568 targets to AKT3 in HCC to regulate stemness and chemosensitivity. **a**. Heatmap summarizing the differentially expressed genes in miR-568 overexpressed SNU-449 cells. **b**. KEGG analysis for enriched pathways in miR-568 overexpressed SNU-449 cells. **c**. The potential binding between miR-568 and ATK3 sequences predicted by TargetScan. **d**. The relative luciferase activities in 293 T cells co-transfected with miR-568 mimics or miR-NC and luciferase reporter vectors AKT3-WT or AKT3-Mut. **e**. Regression curve with respect to expression of miR-568 and AKT3. **f**. Expression of AKT3 mRNA after knockdown or overexpression of miR-568. **g**. Expression of AKT3 protein after knockdown or overexpression of miR-568. **h**. Expression of stemness biomarkers in SNU-449 cells co-transfected with miR-568 mimics or miR-568 mimics NC and AKT3 or AKT3-vec. **i.** Sphere formation capacities of SNU-449 cells after co-transfection with miR-568 mimics or miR-568 mimics NC and AKT3 or AKT3-vec. **j.** Analysis of SNU-449 cell viability after co-transfection with miR-568 mimics or miR-568 mimics NC and AKT3 or AKT3-vec under different concentrations of 5-Fu. ^**^P < 0.01

Given that SNU-449 cells had comparatively higher level of LINC00680, and that LINC00680 was negatively correlated with the level of miR-568 in clinical HCC tissues (Supplementary Figure S2a), we subsequently performed with gene chip analysis to examine gene expression profiling of SNU-449 cells transfected with miR-568 mimic and negative control, so as to further elucidate the potential mechanism of miR-568 in human HCC cells (Fig. 5a). Kyoto Encyclopedia of Genes and Genomes (KEGG) pathway analysis of the gene chip expression data showed that the most significantly enriched pathway upon miR-568 mimic transfection was PI3K-AKT signaling pathway (Fig. 5b). Of which, AKT3, a well-recognized oncogenic gene in HCC [16, 17], was the significantly downregulated genes by transfection of miR-568 (*P* < 0.01) based on the expression profiling data. In addition, it was predicted that there existed a potential binding between miR-568 and ATK3 sequences using bioinformatics online database TargetScan (Fig. 5c). A significant positive correlation between LINC00680 and AKT3 was also seen in HCC specimen (Supplementary Figure S2b). We thus hypothesized that miR-568 might act as an HCC suppressor via specifically targeting binding and degradation of AKT3. This binding relationship was verified in a dual-luciferase reporter gene assay, in which it was found that co-transfection with miR-568 and WT 3’UTR of AKT3 remarkably decreased firefly luciferase activity, whereas which was not affected by co-transfection of miR-568/AKT3 3’UTR mutant, miR-568 NC/AKT3 3’UTR WT, or miR-568 NC/AKT3 3’UTR mutant (Fig. 5d). Additionally, transfection with miR-568 significantly reduced the expression of AKT3 both at mRNA and protein levels, while transfection with miR-568 inhibitor produced an opposite result (Fig. 5f, g).

To confirm the role of AKT3 in the regulation of HCC stemness and chemosensitivity by miR-568 in HCC cells, we restored AKT3 expression in miR-568 mimics transfected cells. The results showed that AKT3 overexpression in HCC cells alleviated the inhibitory effects of miR-568 mimic on stemness markers (CD133, OCT4, NANOG, and SOX-2) (Fig. 5h, Supplementary Figure S3a). Likewise, AKT3 overexpression also blunted miR-568 mimics triggered sphere formation inhibition (Fig. 5i, Supplementary Figure S3b). Moreover, we found that that miR-568 mimics enhanced chemosensitivity of HCC cells to 5-Fu was effectively reversed by AKT3 overexpression (Fig. 5j, Supplementary Figure S3c). These above results suggested that AKT3 was a direct target of miR-568 in HCC, and miR-568 acted as a tumor suppressor through targeting AKT3 mRNA for degradation.

### MiR-568/AKT3/mTOR signaling mediates LINC00680 induced HCC stemness and chemoresistance

**Fig. 6:**
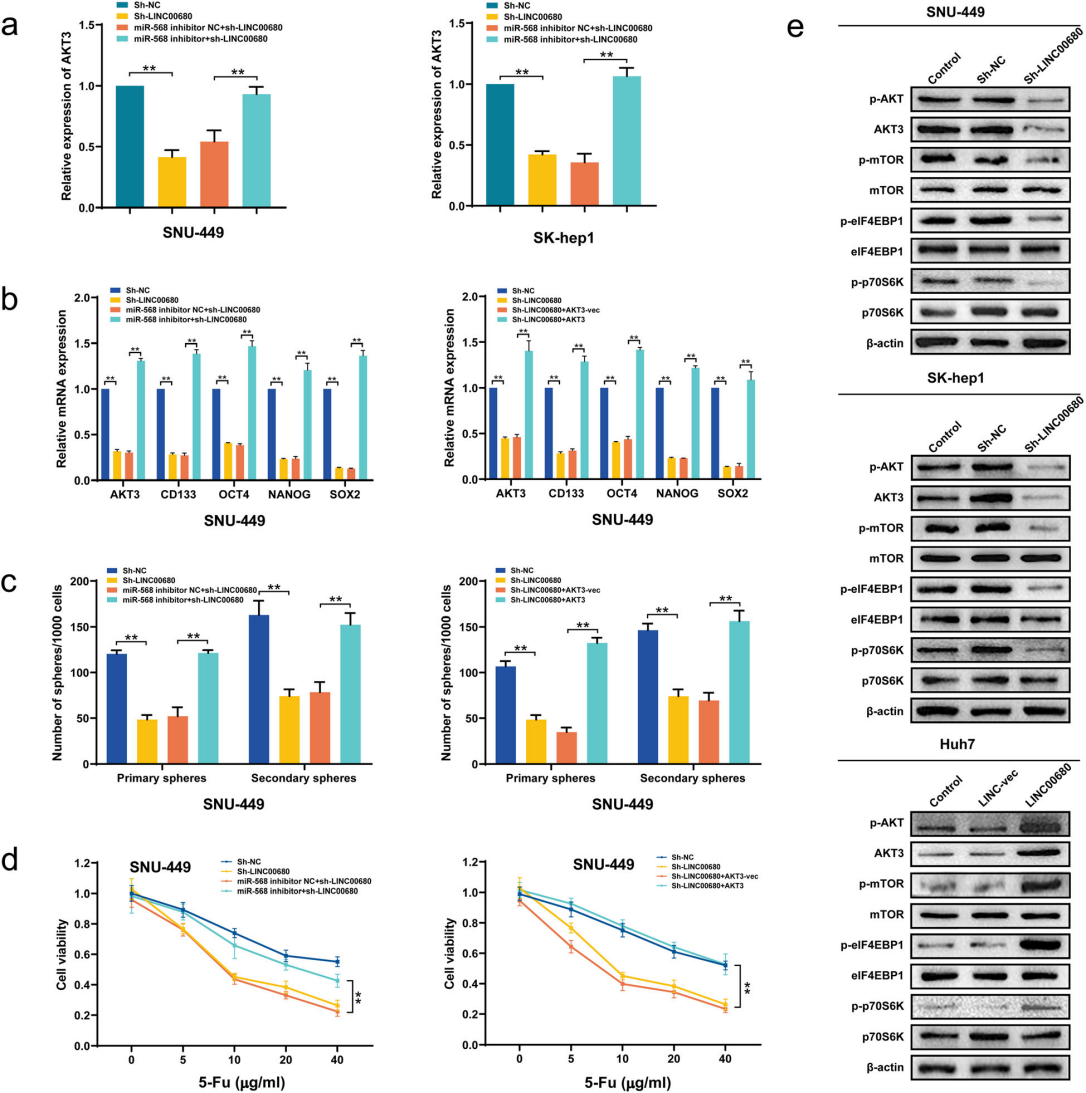
The LINC00680-miR-568-AKT3 axis regulates stemness properties and chemosensitivity in HCC cells. **a**. Expression of AKT3 in SNU-449 cells and SK-hep1 co-transfected with miR-568 inhibitors or miR-568 inhibitors NC and sh-LINC00680 or sh-LINC00680 NC. **b**. Expression of stemness markers in SNU-449 cells co-transfected with miR-568 inhibitor or miR-568 inhibitor NC and Sh-LINC00680 or sh-LINC00680 NC (left); Expression of cellular stemness biomarkers in SNU-449 co-transfected with Sh-LINC00680 or sh-LINC00680 NC and AKT3 or AKT3-vec (right). **c**. Sphere-forming capacities of SNU-449 cells co-transfected with miR-568 inhibitor or miR-568 inhibitor NC and sh-LINC00680 or Sh-LINC00680 NC (left); Sphere-forming capacities of SNU-449 cells co-transfected with sh-LINC00680 or sh-LINC00680 NC and AKT3 or AKT3-vec. (right) **d**. Cell viability analysis for SNU-449 cells co-transfected with Sh-LINC00680 or Sh-LINC00680 NC and miR-568 inhibitor or miR-568 inhibitor NC following treatment by different concentrations of 5-Fu (left); Cell viability analysis for SNU-449 cells co-transfected with sh-LINC00680 or sh-LINC00680 NC and AKT3 or AKT3-vec following treatment by different concentrations of 5-Fu (right). **e**. Expression of total and phosphorylated AKT3, mTOR, eIF4ebp1, p70S6K following manipulation of LINC00680 in HCC cell lines. ^**^P < 0.01

Subsequently, we further explored the potential significance of miR-568/AKT3 axis in LINC00680 mediated HCC tumorigenesis via rescue assays. Although LINC00680/miR-568/AKT3 signaling seemed not affected by 5-Fu intervention in the mouse xenograft model, miR-568 and AKT3 were significantly upregulated and downregulated respectively following LINC00680 knockdown (Supplementary Figure S4a, b). As shown in Fig. 6a, co-transfection of miR-568 inhibitor significantly reversed the inhibition of AKT3 expression induced by LINC00680. Furthermore, miR-568 inhibitor or AKT overexpression successfully reversed the downregulation of stemness markers CD133, OCT4, NANOG, and SOX-2 caused by LINC00680 knockdown (Fig. 6b, Supplementary Figure S5a, b). The reverse of miR-568 inhibitor or AKT overexpression on LINC00680 knockdown mediated stemness decrease was further confirmed in the sphere formation assays (Fig. 6c, Supplementary Figure S5c, d). In addition, through CCK8 analysis, we also observed that miR-568 inhibitor or AKT overexpression significantly abrogated LINC00680 knockdown produced enhancement of chemosensitivity to 5-Fu (Fig. 6d, Supplementary Figure S5e, f). Mounting data demonstrate that AKT-mTOR and their downstream target molecules such as elF4EBP1 and p70S6K act as positive regulators for HCC progression [18–21]. In compatible with this, we herein found that LINC00680 knockdown significantly suppressed the phosphorylation of mTOR, elF4EBP1, and p70S6K, whereas overexpression of LINC00680 enhanced their phosphorylation (Fig. 6e). Taken together, these results suggested that miR-568/AKT3/mTOR signaling axis mediated the oncogenic effects of LINC00680 in HCC.

## Discussion

To date, the conventional chemotherapy treatments in patients with HCC are still not ideal due to the frequently occurring chemoresistance [22, 23]. A growing number of studies have showed that the exiting of cancer stem cells and their enrichment during chemotherapy is closely associated with chemotherapy failure and HCC relapse [24–26]. Thus an effective strategy to sensitize HCC to chemotherapeutic agents was to inhibit HCC stemness properties.

An increasing number of studies have demonstrated the critical modulating role of lncRNAs in tumor stemness phenotype as well as chemosensitivity [27, 28]. In the current study, we for the first time systematically explored the potential significance of LINC00680 in HCC stemness properties and sensitivity to conventional HCC chemotherapeutic drug 5-Fu. Our data unveiled a significantly upregulated expression of LINC00680 in HCC tissues and cell lines, when compared with the adjacent normal liver tissues and normal liver cell line L02, respectively. So far, the functional significance of the long intergenic lncRNA LINC00680 in HCC is completely unknown. Actually, currently there exists only one study reporting its oncogenic role in glioma [12]. In our study, it was found that LINC00680 was positively associated with HCC tumor size and stage, vascular invasion, and poor survival prognosis, suggesting that LINC00680 might play an important role in HCC malignancy. Of note, our subsequent data confirmed that LINC00680 can significantly enhanced HCC stemness, as evidenced by increased primary and secondary sphere formation and HCC markers. In addition, LINC00680 also decrease HCC chemosensitivity to 5-Fu both in vitro and in vivo.

Based on the above findings, we further analyzed the underlying molecular mechanism by which LINC00680 modulated HCC stemness and chemosensitivity. Mounting information supports that sequestering miRNA to regulate mRNA expression via acting as a competing endogenous RNA (ceRNA) represents a commonly used mechanism by lncRNAs to affect HCC progression [29, 30]. Bioinformatics analysis suggests that LINC00680 has putative binding sites for miR-568, which was further confirmed by our luciferase reporter, RIP, and RNA pull-down assays. In addition, manipulation of LINC00680 led to a correspondingly reverse alteration of miR-568 expression. Moreover, a significant downregulation of miR-568 was seen in HCC tumor tissues. All these data imply that LINC00680 is very likely to exert its biological function via sponging miR-568.

In view of that the biological functions of miR-568 in tumor diseases, including HCC, are completely unknown, we then determined whether miR-568 per se affected HCC progression and found that miR-568 could significantly weakened HCC stemness and correspondingly enhanced chemosensitivity to 5-Fu. Using microarray chip assay, it was found that PI3K-AKT signaling pathway was remarkably affected upon miR-568 overexpression, coupled with downregulation of AKT3. Given the well-recognized tumor-promoting role of AKT3 as reported previously [16, 31, 32], we thus reckoned that miR-568 might function as an HCC suppressor in HCC via targeting degradation of AKT3. This hypothesis was strongly supported by our experimental results, which, deriving from luciferase reporter assay, loss- and gain-of-function experiments, and expression correlation analysis in HCC tissues, demonstrated the miR-568 indeed could direct influence AKT3 level in HCC cells. Moreover, we also found that miR-568 weakened HCC stemness and enhanced chemosensitivity to 5-Fu were effectively reversed by AKT3 overexpression.

The mammalian target of rapamycin (mTOR) is aberrantly phosphorylated and activated by PI3K-AKT signaling in many human malignancies, including HCC, and plays a critical role in HCC oncogenesis [19, 33, 34]. mTOR is a serine/threonine protein kinase and exist as two distinct forms: mTORC1 and mTORC2 [35, 36]. Rapamycin-sensitive mTORC1 promote HCC growth and progression via directly targeting phosphorylation and activation of ribosomal protein S6 kinase (p70S6K) and eukaryotic translation initiation factor 4E-binding protein 1 (eIF4EBP1) [37]. Moreover, Inhibition of mTORC1 by rapamycin has been reported to sensitive HCC cells to a variety of chemotherapeutic agents, such as cisplatin, doxorubicin, and histone deacetylase inhibitors, etc. [38]. In addition to AKT3, we in this present study also found that mTOR and its downstream target molecules p70S6K and eIF4EBP1 were all phosphorylated and activated by LINC00680 overexpression, whereas knockdown LINC00680 significantly inhibited their phosphorylation, suggesting that mTOR mediated p70S6K and eIF4EBP1 activation might play an integral role for transduction of LINC00680/miR-568/AKT3 signaling and the corresponding modulation of tumor stemness and chemosensitivity in HCC.

## Conclusions

In summary, our study for the first time has confirmed that LINC00680 is a major driver of HCC stemness, which consequently decreased tumor sensitivity to chemotherapeutic agents, such as 5-Fu. Mechanically, it was found that LINC00680 could sponge miR-568, thereby hindering its repression of AKT3 expression. In addition, several PI3K-AKT downstream signaling molecules, such as mTOR p70S6K, eIF4EBP1 were also be modulated following LINC00680 manipulation. Our findings would facilitate our understanding regarding the role of LINC00680 in HCC progression, and more importantly provide a promising intervention target for overcoming HCC chemoresistance.

## Supplementary Information

**Additional file 1: Figure S1.** Expression of stemness-related markers in sh-LINC00680- or sh-NC-transfected SNU-449 (**a**) and SK-hep1(**b**) cells in nude mice after treatment with 5-Fu. ^**^P < 0.01.

**Additional file 2: Figure S2.** Influence of miR568/AKT3 on SK-hep1 cell stemness and chemosensitivity. **a.** Expression of stemness markers in SK-hep1 cells co-transfected with miR-568 mimics or miR-568 mimics NC and AKT3 or AKT-vec. **b.** Sphere formation capacities of SK-hep1 cells co-transfected with miR-568 mimics or miR-568 mimics NC and AKT3 or AKT-vec. **c.** Cell viability analysis for SNU-449 cells co-transfected with miR-568 mimics or miR-568 mimics NC and AKT3 or AKT-vec following treatment by different concentrations of 5-Fu. ^**^P < 0.01.

**Additional file 3: Figure S3.** A negative correlation analysis between the levels of LINC00680 and miR-568 (**a**), and a positive analysis between between LINC00680 and AKT3 (**b**) in HCC tissues.

**Additional file 4: Figure S4.** Expression of LNC00680, miR-568, and AKT3 in sh-LINC00680- or sh-NC-transfected SNU-449 (**a**) and SK-hep1(**b**) cells in nude mice after treatment with 5-Fu. ^**^P < 0.01.

**Additional file 5: Figure S5.** Implication of miR-568/AKT3 in LINC00680 mediated increases of stemness and chemoresistance in SK-hep1 cells. **a.** Expression of cell stemness markers in SK-hep1 co-transfected with sh-LINC00680 or sh-LINC00680 NC and miR-568 inhibitor or miR-568 inhibitor NC. **b.** Expression of stemness markers in SK-hep1 cells co-transfected with sh-LINC00680 or sh-LINC00680 NC and AKT3 or AKT3-vec. **c.** Sphere formation capacities of SK-hep1 cells co-transfected with sh-LINC00680 or sh-LINC00680 NC and miR-568 inhibitor or miR-568 inhibitor NC. **d.** Sphere formation capacities of SK-hep1 cells co-transfected with sh-LINC00680 or sh-LINC00680 NC and AKT3 or AKT3-vec. **e.** Cell viability analysis of SNU-449 co-transfected with sh-LINC00680 or sh-LINC00680 NC and miR-568 inhibitor or miR-568 inhibitor NC after treatment by different concentrations of 5-Fu. **f.** Cell viability of SNU-449 cells co-transfected with sh-LINC00680 or sh-LINC00680 NC and AKT3 or AKT3-vec after treatment by different concentrations of 5-Fu. ^**^P < 0.01.

**Additional file 6: Table S1.** The sequences of primers for RT-qPCR.

## Data Availability

The datasets used and/or analysed during the current study are available from the corresponding author on reasonable request.

## Abbreviations

HCC: Hepatocellular carcinoma
RIP: RNA binding protein immunoprecipitation
qRT-PCR: Quantitative real time polymerase chain reaction
OS: Overall survival
5-Fu: 5-fluorouracil
ceRNA: Competing endogenous RNAs
CSC: Cancer stem cell
