# Supplementary figures and images for: LINC00680 enhances hepatocellular carcinoma stemness behavior and chemoresistance by sponging miR-568 to upregulate AKT3
Source: J Exp Clin Cancer Res. 2021 Jan 26;40:45. doi: 10.1186/s13046-021-01854-5 (PMC7836199; doi:10.1186/s13046-021-01854-5)

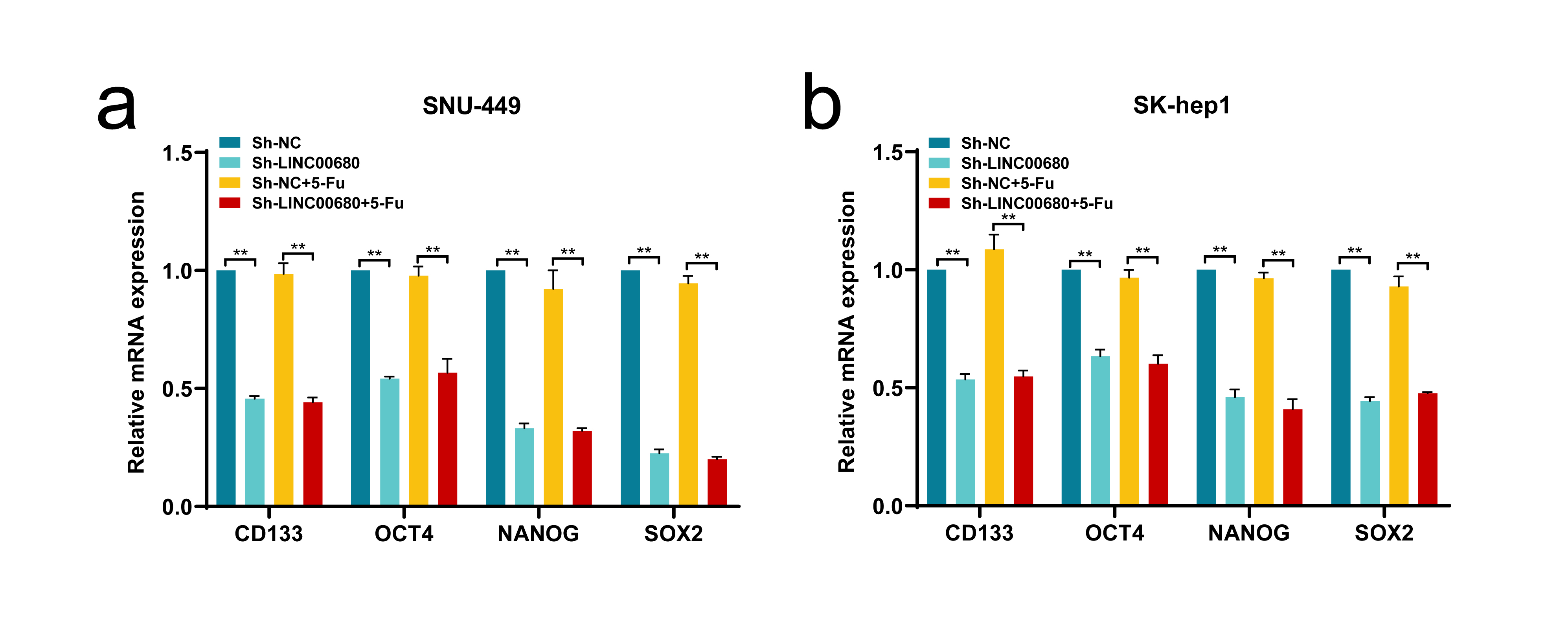

Supplement: Supplementary file 1 — Additional file 1: Figure S1. Expression of stemness-related markers in sh-LINC00680- or sh-NC-transfected SNU-449 (a) and SK-hep1(b) cells in nude mice after treatment with 5-Fu. **P < 0.01. [file 13046_2021_1854_MOESM1_ESM.jpg]

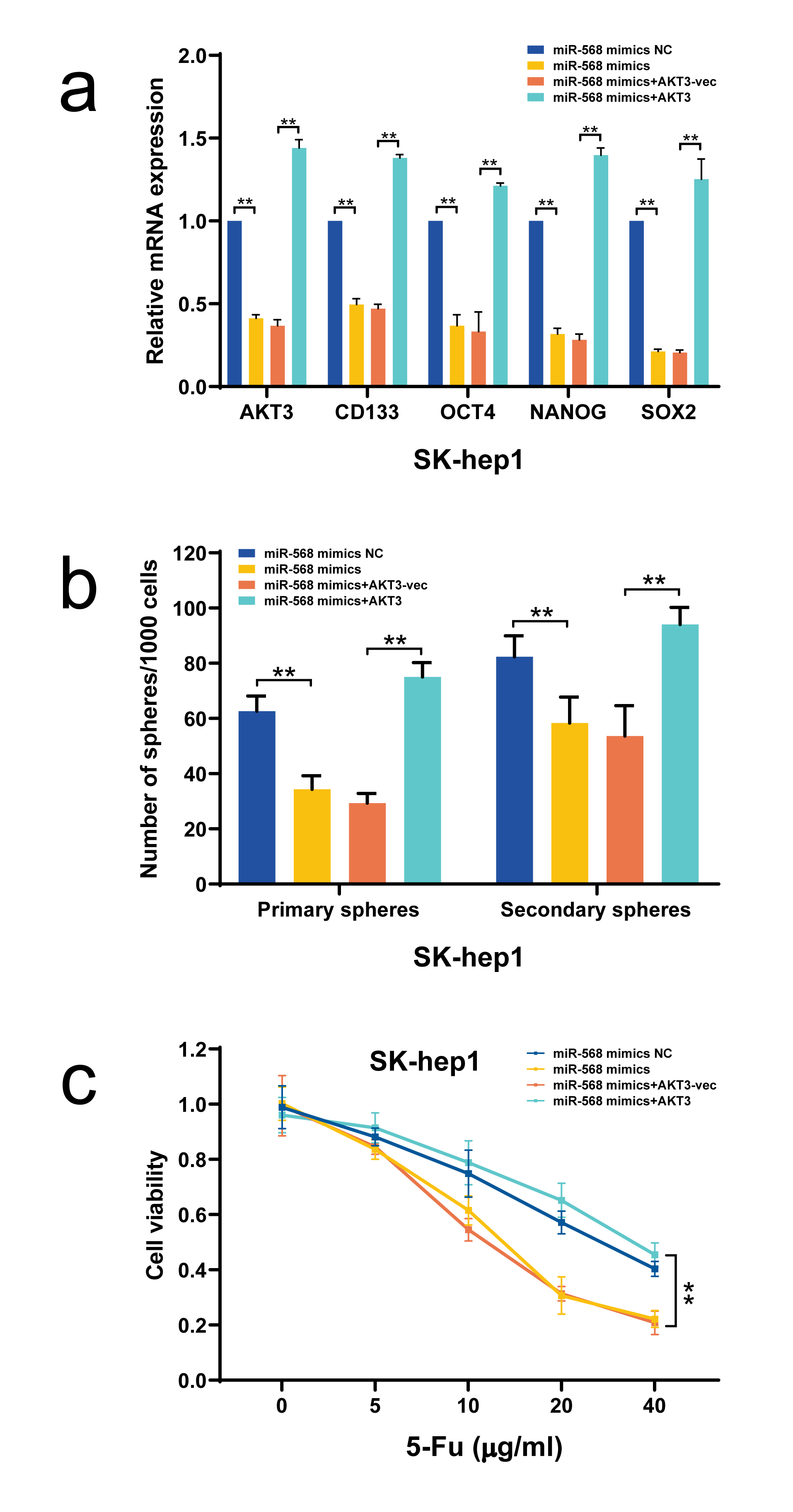

Supplement: Supplementary file 2 — Additional file 2: Figure S2. Influence of miR568/AKT3 on SK-hep1 cell stemness and chemosensitivity. a. Expression of stemness markers in SK-hep1 cells co-transfected with miR-568 mimics or miR-568 mimics NC and AKT3 or AKT-vec. b. Sphere formation capacities of SK-hep1 cells co-transfected with miR-568 mimics or miR-568 mimics NC and AKT3 or AKT-vec. c. Cell viability analysis for SNU-449 cells co-transfected with miR-568 mimics or miR-568 mimics NC and AKT3 or AKT-vec following treatment by different concentrations of 5-Fu. **P < 0.01. [file 13046_2021_1854_MOESM2_ESM.jpg]

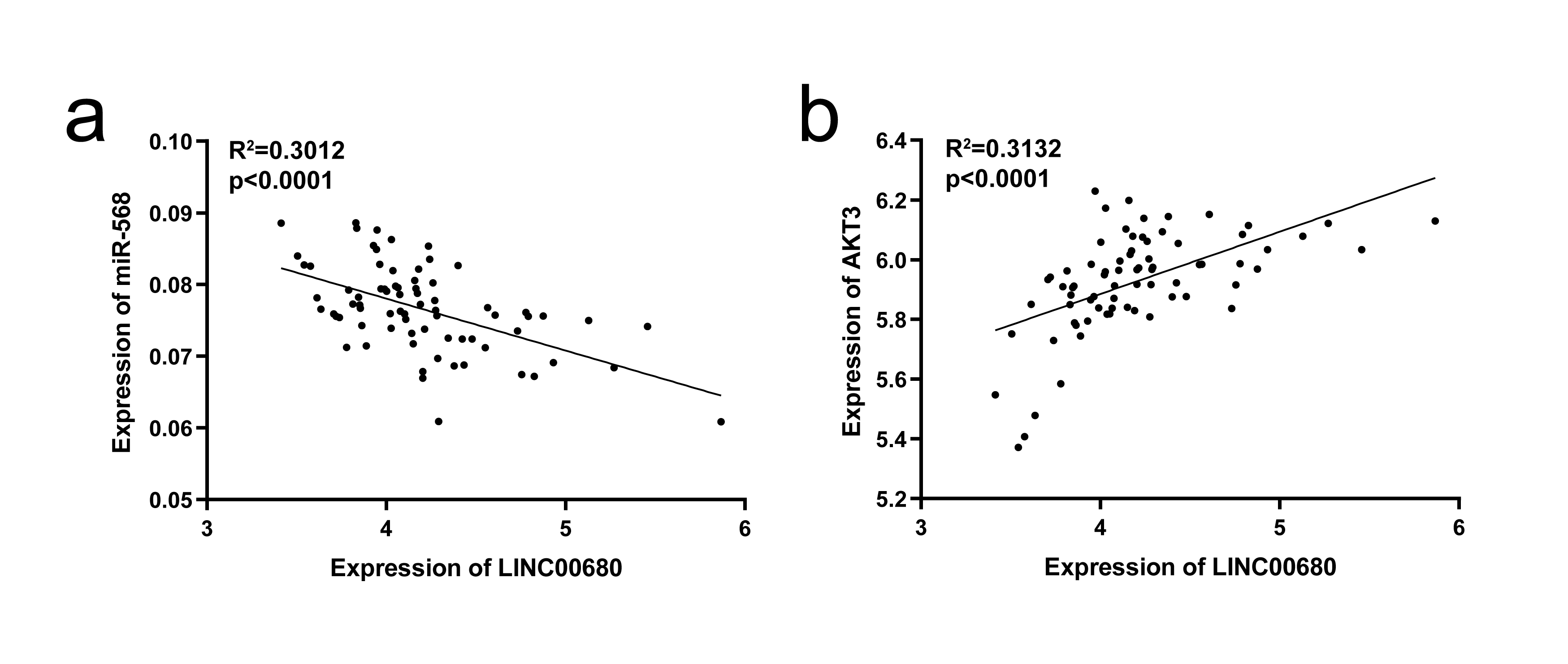

Supplement: Supplementary file 3 — Additional file 3: Figure S3. A negative correlation analysis between the levels of LINC00680 and miR-568 (a), and a positive analysis between between LINC00680 and AKT3 (b) in HCC tissues. [file 13046_2021_1854_MOESM3_ESM.jpg]

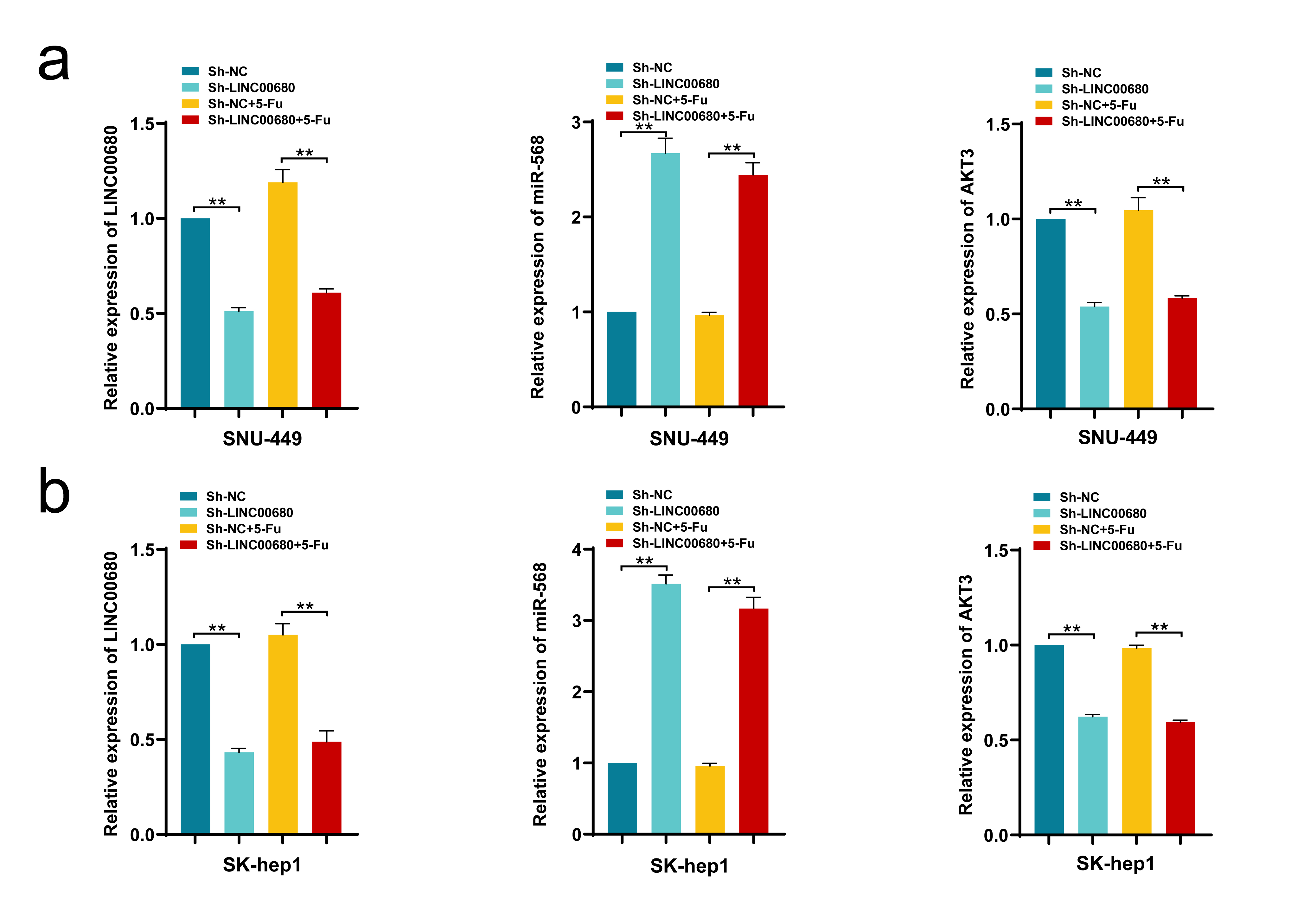

Supplement: Supplementary file 4 — Additional file 4: Figure S4. Expression of LNC00680, miR-568, and AKT3 in sh-LINC00680- or sh-NC-transfected SNU-449 (a) and SK-hep1(b) cells in nude mice after treatment with 5-Fu. **P < 0.01. [file 13046_2021_1854_MOESM4_ESM.jpg]

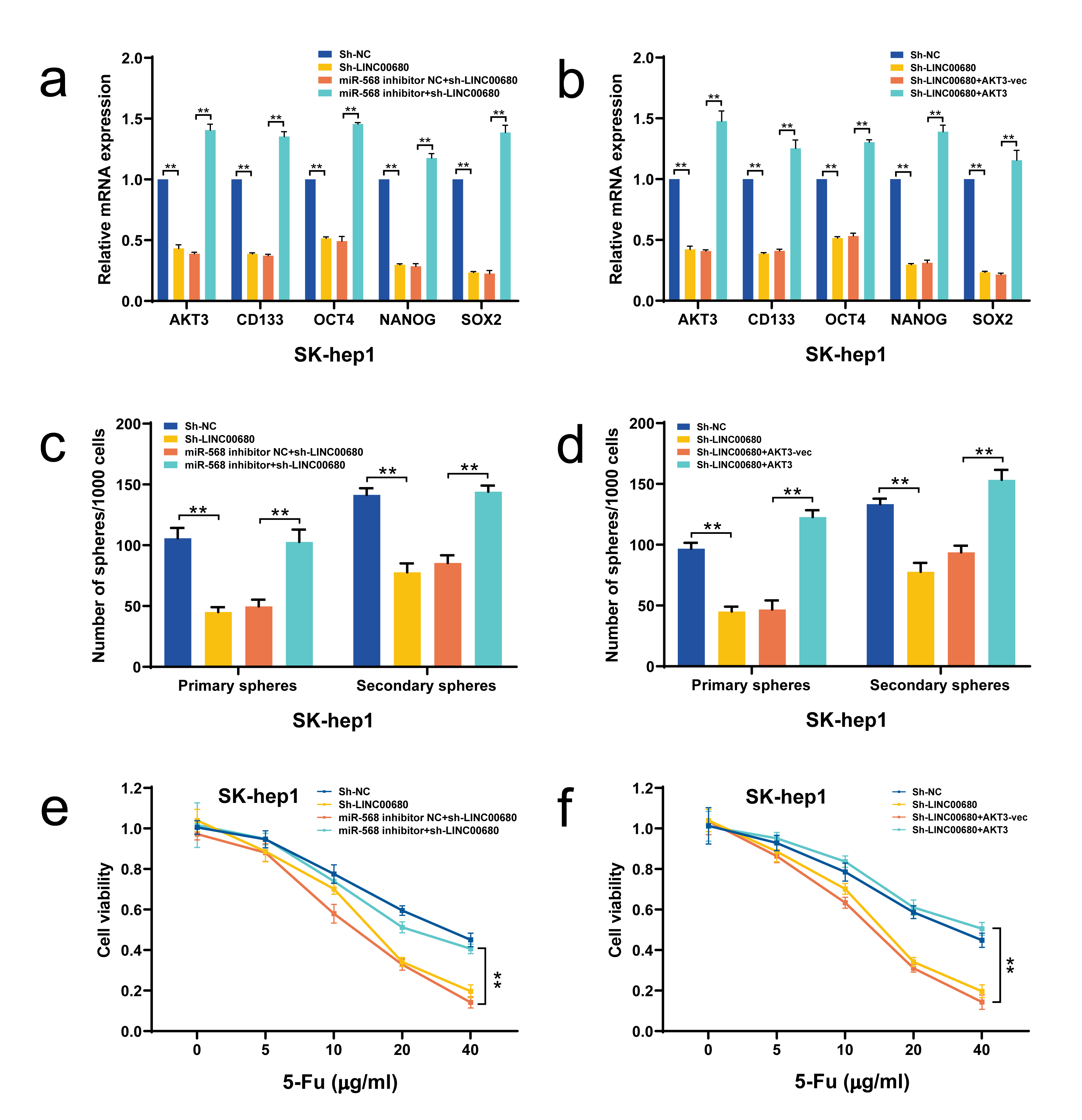

Supplement: Supplementary file 5 — Additional file 5: Figure S5. Implication of miR-568/AKT3 in LINC00680 mediated increases of stemness and chemoresistance in SK-hep1 cells. a. Expression of cell stemness markers in SK-hep1 co-transfected with sh-LINC00680 or sh-LINC00680 NC and miR-568 inhibitor or miR-568 inhibitor NC. b. Expression of stemness markers in SK-hep1 cells co-transfected with sh-LINC00680 or sh-LINC00680 NC and AKT3 or AKT3-vec. c. Sphere formation capacities of SK-hep1 cells co-transfected with sh-LINC00680 or sh-LINC00680 NC and miR-568 inhibitor or miR-568 inhibitor NC. d. Sphere formation capacities of SK-hep1 cells co-transfected with sh-LINC00680 or sh-LINC00680 NC and AKT3 or AKT3-vec. e. Cell viability analysis of SNU-449 co-transfected with sh-LINC00680 or sh-LINC00680 NC and miR-568 inhibitor or miR-568 inhibitor NC after treatment by different concentrations of 5-Fu. f. Cell viability of SNU-449 cells co-transfected with sh-LINC00680 or sh-LINC00680 NC and AKT3 or AKT3-vec after treatment by different concentrations of 5-Fu. **P < 0.01. [file 13046_2021_1854_MOESM5_ESM.jpg]
