# Supplementary material for: LINC00680 enhances hepatocellular carcinoma stemness behavior and chemoresistance by sponging miR-568 to upregulate AKT3
Source: J Exp Clin Cancer Res. 2021 Jan 26;40:45. doi: 10.1186/s13046-021-01854-5 (PMC7836199; doi:10.1186/s13046-021-01854-5)
Supplement: Supplementary file 6 — Additional file 6: Table S1. The sequences of primers for RT-qPCR. [file 13046_2021_1854_MOESM6_ESM.docx]

**Supplementary table 1**

**Table. S1 The sequences of primers for RT-qPCR**

| cDNA | Primers | Sequences |
| --- | --- | --- |
| GAPDH | forward | 5'- CTGCCAACGTGTCAGTGGTG-3' |
|  | reverse | 5'-TCAGTGTAGCCCAGGATGCC-3' |
| U6 | forward | 5'-CGCAAGGATGACACGCAAATTC-3' |
|  | reverse | 5'-GTGCAGGGTCCGAGGT-3' |
| AKT3 | forward | 5'-TGGATGCCTCTACAACCCATCA-3' |
| LINC00680  CD133  OCT4  NANOG  SOX-2 | reverse  forward  reverse  forward  reverse  forward  reverse  forward  reverse  forward  reverse | 5'-TGTGTGCCACTTCATCCTTTGC-3'  5'-CCATCGACTGGCTCATCACAA-3'  5'-GGGGCAAGGCAAATCAATACC-3'  5'-ACACTACCAAGGACAAGGCG-3'  5'-TCTCCAACGCCTCTTTGGTC-3'  5'-CAAAGCAGAAACCCTCGTGC-3'  5'-AACCACACTCGGACCACATC-3'  5'-GATGCCTCACACGGAGACTG-3'  5'-TTGACCGGGACCTTGTCTTC-3'  5'-AGGATAAGTACACGCTGCCC-3'  5'-TTCATGTGCGCGTAACTGTC-3' |
